# Age- and sex-based normal reference ranges of the cardiac time intervals: the Copenhagen City Heart Study

**DOI:** 10.1007/s00392-023-02269-2

**Published:** 2023-07-31

**Authors:** Alia Saed Alhakak, Flemming Javier Olsen, Kristoffer Grundtvig Skaarup, Mats Christian Højbjerg Lassen, Niklas Dyrby Johansen, Peter Godsk Jørgensen, Ulrik Abildgaard, Gorm Boje Jensen, Peter Schnohr, Peter Søgaard, Rasmus Møgelvang, Tor Biering-Sørensen

**Affiliations:** 1 Cardiovascular Non-Invasive Imaging Research Laboratory, Department of Cardiology, Herlev and Gentofte Hospital, University of Copenhagen, Gentofte Hospitalsvej 1, Hellerup, 2900 Copenhagen, Denmark; 2 The Copenhagen City Heart Study, Bispebjerg and Frederiksberg Hospital, University of Copenhagen, Copenhagen, Denmark; 3 Department of Cardiology, Aalborg University Hospital, Aalborg, Denmark; 4 Department of Clinical Medicine, University of Aalborg, Aalborg, Denmark; 5 Department of Cardiology, The Heart Center, Rigshospitalet, University of Copenhagen, Copenhagen, Denmark; 6 Institute of Clinical Medicine, Faculty of Health and Medical Sciences, University of Copenhagen, Copenhagen, Denmark; 7 Cardiovascular Research Unit, University of Southern Denmark, Odense, Denmark; 8 Department of Biomedical Sciences, Faculty of Health and Medical Sciences, University of Copenhagen, Copenhagen, Denmark

**Keywords:** Cardiac time intervals, Normal values, General population, Cardiac cycle, TDI-echocardiography

## Abstract

**Background:**

Color tissue Doppler imaging (TDI) M-mode can be used to measure the cardiac time intervals including the isovolumic contraction time (IVCT), the left ventricular ejection time (LVET), the isovolumic relaxation time (IVRT), and the combination of all the cardiac time intervals in the myocardial performance index (MPI) defined as [(IVCT + IVRT)/LVET]. The aim of this study was to establish normal age- and sex-based reference ranges for the cardiac time intervals.

**Methods and results:**

A total of 1969 participants free of cardiovascular diseases and risk factors from the general population with limited age range underwent an echocardiographic examination including TDI. The median age was 46 years (25th–75th percentile: 33–58 years), and 61.5% were females. In the entire study population, the IVCT was observed to be 40 ± 10 ms [95% prediction interval (PI) 20–59 ms], the LVET 292 ± 23 ms (95% PI 248–336 ms), the IVRT 96 ± 19 ms (95% PI 59–134 ms) and MPI 0.47 ± 0.09 (95% PI 0.29–0.65). All the cardiac time intervals differed significantly between females and males. With increasing age, the IVCT increased in females, but not in males. The LVET did not change with age in both sexes, while the IVRT increased in both sexes with increasing age. Furthermore, we developed regression equations relating the heart rate to the cardiac time intervals and age- and sex-based normal reference ranges corrected for heart rate.

**Conclusion:**

In this study, we established normal age- and sex-based reference ranges for the cardiac time intervals. These normal reference ranges differed significantly with sex.

**Graphical abstract:**

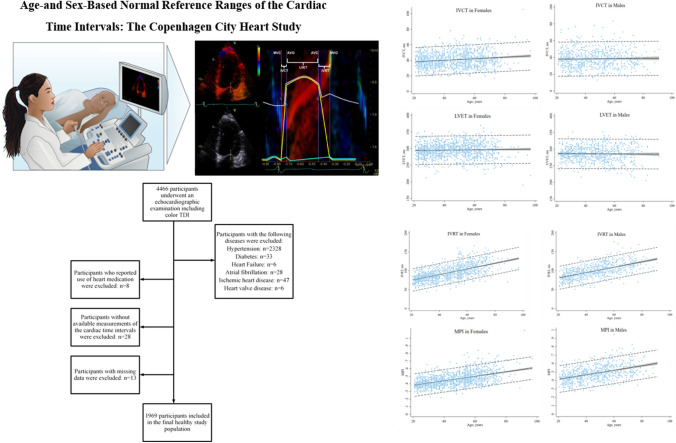

**Supplementary Information:**

The online version contains supplementary material available at 10.1007/s00392-023-02269-2.

## Introduction

The cardiac time intervals include the isovolumic contraction time (IVCT), the left ventricular ejection time (LVET), the isovolumic relaxation time (IVRT), and the combination of all the cardiac time intervals in the myocardial performance index (MPI) defined as [(IVCT + IVRT)/LVET]. In varieties of cardiovascular diseases, the cardiac time intervals have shown to be sensitive markers for diagnosis and prognosis [1–3]. A newer echocardiographic method for obtaining the cardiac time intervals is by applying color tissue Doppler imaging (TDI) M-mode. This method has been shown to be superior to the conventional pulsed-wave Doppler method [4, 5].

It has previously been demonstrated that the cardiac time intervals vary with sex and age [6–8]. Furthermore, the cardiac time intervals are inversely related to heart rate and should be corrected for heat rate [1]. The first normal reference ranges for the systolic cardiac time intervals (IVCT and LVET) corrected for heart rate were established in 1968 [6]. In the meantime, the methods of obtaining the cardiac time intervals and the epidemiology of the general population and cardiovascular diseases have changed. Hence, there is an unmet need to develop new age- and sex-based normal reference ranges for the cardiac time intervals. This is essential to incorporate and use the cardiac time intervals in clinical practice.

The primary aim of this study was to establish age- and sex-based normal reference ranges for the cardiac time intervals. Further, we wanted to develop regression equations relating the heart rate to the cardiac time intervals and age- and sex-based normal reference ranges corrected for heart rate.

## Methods

### Study population

The Copenhagen City Heart Study (CCHS) is a prospective cardiovascular population study of randomly selected participants from the general population in Denmark (clinicaltrials.gov identifier: NCT02993172). Information on this study cohort has been described in detail elsewhere [9]. The current study is an echocardiographic substudy of the fifth CCHS spanning from 2011 to 2015. A total of 4466 participants underwent a health examination including a detailed echocardiographic examination [10]. In order to ensure a healthy study population, participants with the following cardiovascular diseases were excluded: hypertension (*n* = 2328), diabetes (*n* = 33), heart failure (*n* = 6), atrial fibrillation/flutter (*n* = 28), ischemic heart disease (*n* = 47), heart valve disease (*n* = 6) and participants who reported use of heart medication (*n* = 8). Additionally, participants without available measurements of the cardiac time intervals (*n* = 28) were excluded. Finally, participants with missing data (*n* = 13) were excluded. Hence, the final study population consisted of 1969 participants free of cardiovascular diseases and risk factors. This study population was used to develop normal reference ranges for the cardiac time intervals.

The study was conducted according to the 2nd Declaration of Helsinki and approved by the Regional Ethics Committee. All participants gave written informed consent before the examination. All participants attended a general health examination, which included physical examination, self-administered questionnaire and blood samples [9].

### Baseline information

The Danish National Patient Registry was used to collect information on diagnoses using the International Classification of Disease (ICD-8 and ICD-10) codes. The obtained diagnoses included heart valve disease (aortic stenosis/regurgitation or mitral stenosis/regurgitation), atrial fibrillation/flutter, heart failure, ischemic heart disease, diabetes mellitus and hypertension. Definitions of ischemic heart disease, diabetes mellitus and hypertension have previously been described in detail [10].

### Echocardiography

Echocardiography was performed using Vivid 9 ultrasound systems (GE Healthcare, Horten, Norway) by experienced sonographers. The echocardiograms were stored and analyzed offline with commercially available software (EchoPac version 113.15, GE Healthcare, Horten Norway) [10].

Measures of left ventricular (LV) dimensions were performed in the parasternal long-axis view at end-diastole. The LV dimensions included interventricular septal diameter, LV internal diameter and LV posterior wall diameter. LV mass index (LVMI) was estimated by dividing LV mass with the body surface area. LV hypertrophy was defined as LVMI ≥ 96 g/m^2^ for females and ≥ 116 g/m^2^ for males. LV ejection fraction (LVEF) was estimated by the Simpson’s biplane method [11].

Two-dimensional speckle tracking echocardiography was performed in the three apical views by using an automated function that defined a region of interest (ROI). This has previously been described in detail [10]. Global longitudinal strain (GLS) was calculated as an average peak strain from the three apical views.

The left atrial (LA) volume was measured using the biplane area-length method and indexed to body surface area to create left atrial volume index (LAVI) [11]. Pulsed-wave Doppler was used to record the mitral inflow between the tips of the mitral valve leaflets. The peak velocity of early (E) and atrial (A) diastolic filling and deceleration time of the E-wave were measured, and the E/A ratio was calculated.

The color TDI was used to obtain the peak systolic (*s*′), early diastolic (*e*′) and late diastolic (*a*′) velocities. The average values of *e*′ were used to calculate *E*/*e*′.

### Tissue Doppler imaging

**Fig. 1:**
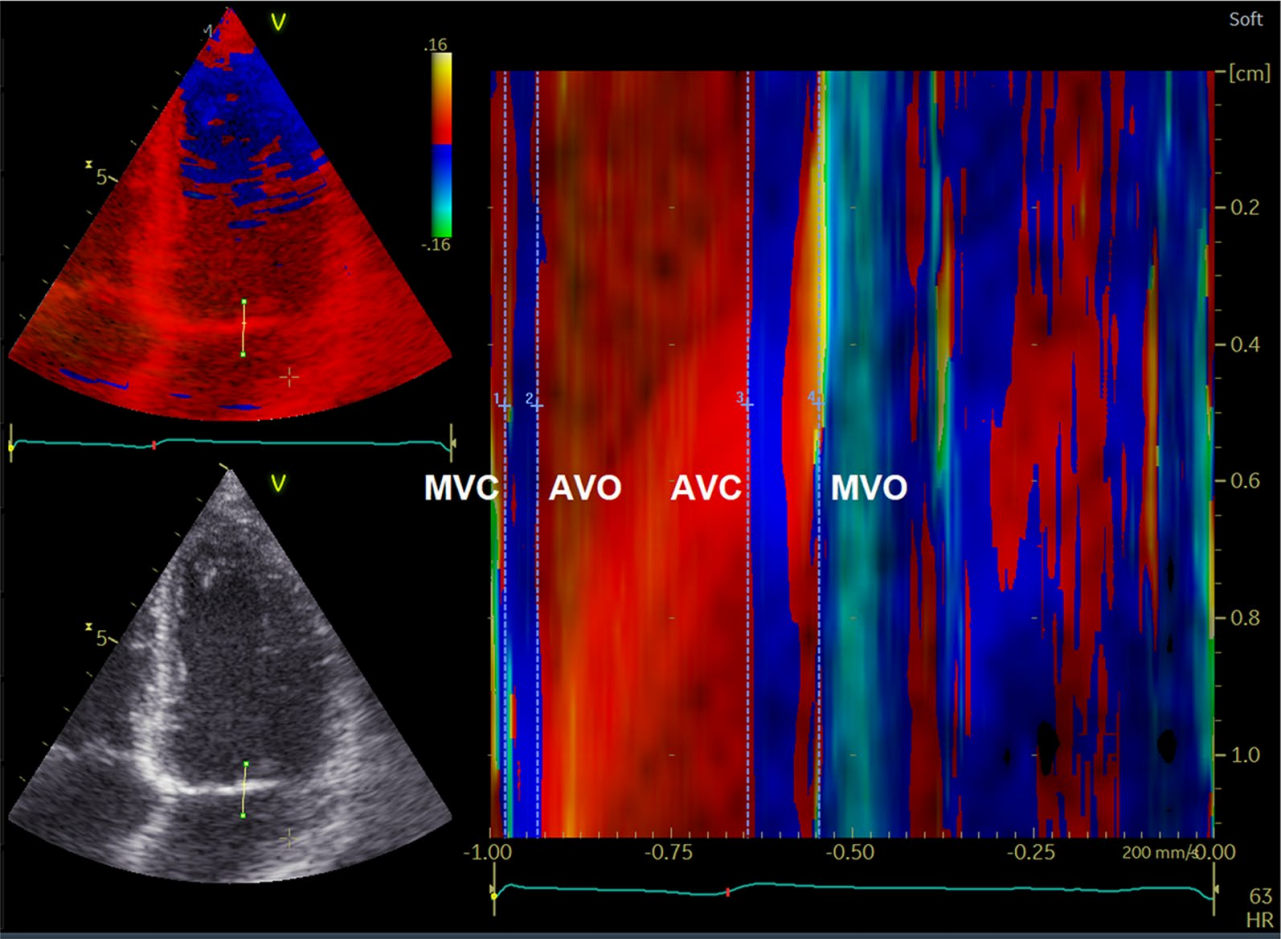
The cardiac time intervals assessed by color tissue Doppler imaging (TDI) M-mode through the mitral leaflet. *AVO* aortic valve opening, *AVC* aortic valve closing, *MVC* mitral valve closing, *MVO* mitral valve opening

The cardiac time intervals were measured from the color TDI 4-chamber view at the highest possible frame rate (median frame rate: 162 frames per second [25th–75th percentile: 161–166 frames per second]). A 2–4 cm straight M-mode line was placed through the septal half of the anterior mitral valve leaflet (Fig. 1).

The mitral valve closure (MVC) was determined by the color shift from blue/turquoise to red at end-diastole. The aortic valve opening (AVO) was determined by the color shift from blue to red at the beginning of the systole. The aortic valve closure (AVC) was determined by the color shift from red to blue at end-systole. The mitral valve opening (MVO) was determined by the color shift from red orange to yellow.

The IVCT was defined as the time interval from the MVC to AVO, the LVET was defined as the time interval from the AVO to AVC, and the IVRT was defined as the time interval from the AVC to MVO. This method has previously been described in detail and a validation of this method has been performed [4, 5, 12].

### Statistics

The statistical analyses were conducted using STATA SE version 15.1 (StataCorp, College Station, Texas). Histograms and QQ-plots were used to assess for normal distribution. Continuous Gaussian distributed variables were compared using Student *t* test and reported as mean ± SD. Kruskal–Wallis test was used for comparing continuous non-Gaussian distributed variables and reported as medians with 25th–75th percentiles. Categorical variables were compared using Chi-square test and reported as percentages.

*p* values for trend were calculated using linear regression for continuous distributed variables and by Chi-square test for trend for proportions.

Normal reference ranges for the cardiac time intervals were established for the entire study sample. Additionally, to establish age- and sex-based normal reference ranges, the study sample was stratified by sex and by sex and age categories (20–34, 35–49, 50–64, and > 65 years). The normal reference ranges were reported as mean ± SD with corresponding 95% prediction intervals (PI). Prediction plots were generated to illustrate the relationship between the cardiac time intervals and age. Regression equations relating the heart rate to the cardiac time intervals were calculated.

We conducted different sensitivity analyses to determine whether the size of the LAVI, the value of GLS or PALS affected our results. We defined normal LAVI as ≤ 34 mL/m^2^ and abnormal LAVI as > 34 mL/m^2^ [13]. Normal GLS was defined as an absolute value of ≥ 15.8% and abnormal GLS < 15.8% [10]. Normal PALS was defined as a value ≥ 23% and abnormal PALS as < 23% [14]. The definitions of GLS and PALS are based on data from the CCHS [10, 14].

A *p* value < 0.05 in two-sided tests was considered statistically significant.

## Results

### Baseline characteristics

The baseline characteristics for the entire study population and stratified by age categories are shown in Table 1. The participants median age was 46 years (25th–75th percentile: 33–58 years), and 61.5% (*n* = 1211) were females. The mean systolic and diastolic blood pressure were 124 mmHg (25th–75th percentile: 116–131 mmHg) and 74 mmHg (25th–75th percentile: 69–80 mmHg), respectively, and mean heart rate was 63 ± 10 beats per minute. The mean LVEF was 58 ± 5%

**Table 1:** Baseline characteristics of the population stratified by age categories

Age ranges, years	All *n* = 1969	20–34 *n* = 558	35–49 *n* = 566	50–64 *n* = 567	> 65 *n* = 278	*p* value
Demographics						
Age, years	46 (33–58)	28 (25–31)	41 (37–46)	56 (53–60)	71 (67–74)	< 0.001
Female, *n* (%)	1211 (61.5%)	350 (62.7%)	331 (58.5%)	367 (64.7%)	163 (58.6%)	0.11
Clinical charachteristics						
Systolic blood pressure, mmHg	124 (116–131)	122 (114–128)	123 (115–130)	125 (117–132)	128 (121–134)	< 0.001
Diastolic blood pressure, mmHg	74 (69–80)	72 (67–77)	75 (69–81)	76 (71–81)	73 (68–78)	< 0.001
Heart rate, bpm	63 ± 10	63 ± 10	62 ± 10	64 ± 10	67 ± 11	< 0.001
BMI, kg/m^2^	24.3 ± 3.9	23.1 ± 3.2	24.3 ± 3.6	25.2 ± 4.2	24.8 ± 4.5	< 0.001
Smoking status, pack-years	0.0 (0.0–7.5)	0.0 (0.0–1.3)	0.0 (0.0–5.7)	3.3 (0.0–7.5)	0.6 (0.0–19.7)	< 0.001
Physical activity level in leisure time						< 0.001
Sedentary, *n* (%)	99 (5.1%)	18 (3.2%)	38 (6.7%)	29 (5.1%)	14 (5.2%)	
Low, *n* (%)	634 (32.4%)	140 (25.2%)	166 (29.4%)	210 (37.2%)	118 (43.5%)	
Moderate, *n* (%)	1012 (51.8%)	291 (52.3%)	295 (52.3%)	295 (52.3%)	131 (48.3%)	
High, *n* (%)	201 (10.7%)	107 (19.7%)	65 (11.5)	30 (5.3%)	8 (3.0%)	
Laboratory work						
Total cholesterol, mmol/L	5.2 ± 1.1	4.7 ± 0.9	5.1 ± 1.0	5.7 ± 1.0	5.8 ± 1.0	< 0.001
LDL cholesterol, mmol/L	3.0 ± 0.9	2.5 ± 0.8	2.9 ± 0.9	3.3 ± 0.9	3.4 ± 0.9	< 0.001
HDL cholesterol, mmol/L	1.6 ± 0.5	1.6 ± 0.5	1.5 ± 0.4	1.7 ± 0.5	1.7 ± 0.5	< 0.001
Glucose, mmol/L	5.2 ± 0.8	4.9 ± 0.7	5.1 ± 0.8	5.3 ± 0.9	5.5 ± 1.0	< 0.001
Creatinine, mmol/L	74.6 ± 11.4	72.4 ± 11.5	74.6 ± 11.4	75.3 ± 10.6	77.8 ± 12.0	< 0.001
Hemoglobin, mmol/L	8.7 ± 0.7	8.7 ± 0.1	8.7 ± 0.8	8.7 ± 0.7	8.6 ± 0.7	< 0.001
Echocardiography						
LVEF, %	57.5 ± 5.3	57.3 ± 4.5	58.0 ± 5.0	57.4 ± 6.0	57.1 ± 6.0	0.07
GLS, %	− 19.9 ± 2.1	− 19.9 ± 1.9	− 20.0 ± 2.1	− 19.9 ± 2.1	− 19.7 ± 2.5	0.29
LVMI, g/m^2^	79.6 ± 17.6	77.9 ± 16.9	79.7 ± 17.8	80.7 ± 17.1	80.8 ± 19.2	0.038
LAVI, mL/m^2^	23.0 ± 7.0	22.5 ± 6.5	23.0 ± 7.2	23.4 ± 7.0	23.3 ± 7.7	0.13
DT, ms	189.0 ± 40.9	178.5 ± 32.9	183.6 ± 35.6	193.5 ± 41.6	212.4 ± 52.5	< 0.001
*E*/*e*′	6.0 (5.1–7.2)	5.3 (4.7–6.0)	5.7 (5.0–6.5)	6.7 (5.7–7.9)	8.2 (6.6–9.5)	< 0.001
*E*/*A*	1.4 (1.1–1.8)	1.9 (1.6–2.2)	1.5 (1.3–1.8)	1.1 (1.0–1.3)	0.9 (0.8–1.1)	< 0.001

*A* peak transmitral late diastolic inflow velocity, *BMI* body mass index, *DT* deceleration time of early diastolic inflow, *E* peak transmitral early diastolic inflow velocity, *e*′ average peak early diastolic longitudinal mitral annular velocity determined by color TDI, *GLS* global longitudinal strain, *HDL* high-density lipoprotein, *LAVI* left atrial volume index, *LDL* low-density lipoprotein, *LVEF* left ventricular ejection fraction, *LVMI* left ventricular mass index

### Normal reference ranges of the cardiac time intervals

In Table 2, the normal reference ranges including 95% prediction intervals of the cardiac time intervals are shown for the entire study population and stratified by sex. In the entire study population, the IVCT was observed to be 40 ± 10 ms (95% PI 20–59 ms), the LVET 292 ± 23 ms (95% PI 248–336 ms), the IVRT 96 ± 19 ms (95% PI 59–134 ms) and MPI 0.47 ± 0.09 (95% PI 0.29–0.65). All the cardiac time intervals differed significantly between females and males (*p* < 0.001 for all). The IVCT and LVET were significantly longer in females than males, while the IVRT was significantly longer in males than females. Greater values of MPI were observed in males than females.

**Table 2:** Normal values of the cardiac time intervals stratified by sex

Cardiac time intervals	All *n* = 1969	Females *n* = 1211	Males *n* = 758	*p* value for sex difference
IVCT, ms	40 ± 10 (20–59)	41 ± 9 (22–59)	38 ± 10 (18–59)	< 0.001
LVET, ms	292 ± 23 (248–336)	296 ± 22 (253–338)	286 ± 23 (242–330)	< 0.001
IVRT, ms	96 ± 19 (59–134)	94 ± 19 (57–132)	100 ± 19 (62–137)	< 0.001
MPI	0.47 ± 0.09 (0.29–0.65)	0.46 ± 0.09 (0.28–0.64)	0.49 ± 0.09 (0.31–0.67)	< 0.001

*IVCT* isovolumic contraction time, *IVRT* isovolumic relaxation time, *LVET* left ventricular ejection time, *MPI* myocardial performance index

**Fig. 2:**
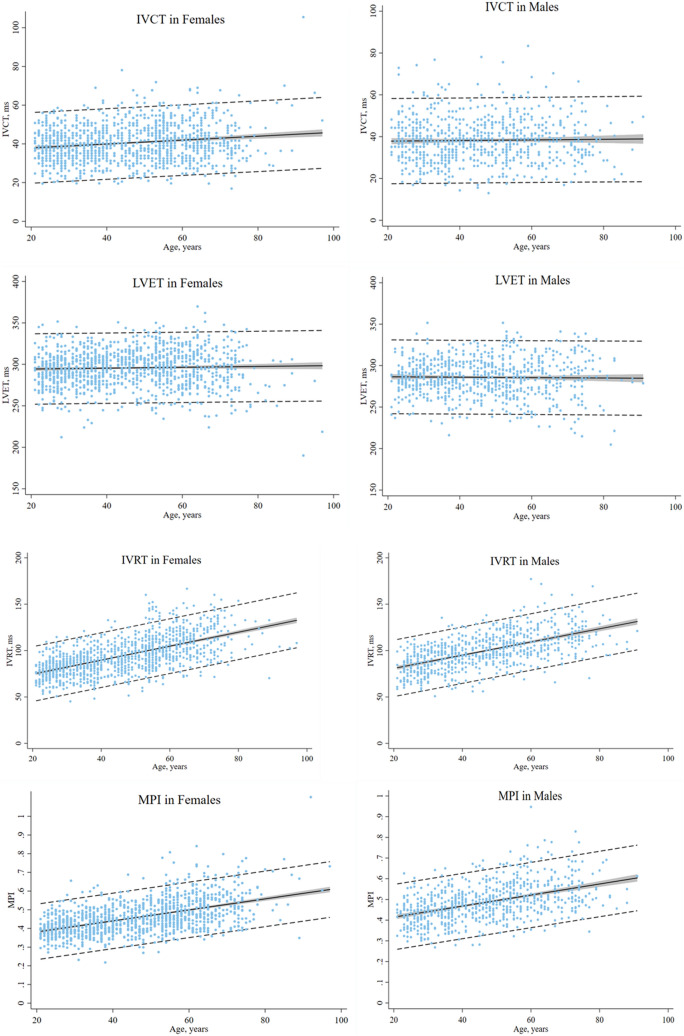
Normal reference ranges of the IVCT, LVET, IVRT and MPI according to sex and age. The regression line is in black. The 95% confidence interval is in gray. The 95% prediction interval is the dotted black line. *IVCT* isovolumic contraction time, *IVRT* isovolumic relaxation time, *LVET* left ventricular ejection time, *MPI* myocardial performance index

Table 3 shows normal reference ranges including 95% PI of the cardiac time intervals stratified by sex and age. Sex modified the relationship between IVCT and age (*p* for interaction = 0.003), such that the IVCT increased significantly across age categories in females (*p* < 0.001); however, the IVCT did not increase significantly across age categories in males (*p* = 0.55). The LVET did not change significantly across age categories in neither females (*p* = 0.08) nor males (0.38). The IVRT increased significantly across age categories in both females (*p* < 0.001) and males (*p* < 0.001). The MPI increased significantly across age categories in both females (*p* < 0.001) and males (*p* < 0.001). Figure 2 illustrates the association between the cardiac time intervals and age in females and males.

**Table 3:** Normal values of the cardiac time intervals across age and sex

Age range, years	Females					Males				
	20–34 *n* = 350	35–49 *n* = 331	50–64 *n* = 367	> 65 *n* = 163	*p* value for trend	20–34 *n* = 208	35–49 *n* = 235	50–64 *n* = 200	> 65 *n* = 115	*p* value for trend
IVCT, ms	38 ± 8 (23–54)	40 ± 9 (22–58)	42 ± 10 (23–61)	42 ± 11 (20–64)	< 0.001	38 ± 11 (17–60)	37 ± 10 (18–57)	39 ± 11 (18–60)	38 ± 10 (19–58)	0.55
LVET, ms	292 ± 20 (253–331)	297 ± 19 (259–335)	299 ± 22 (256–343)	292 ± 27 (240–345)	0.08	286 ± 19 (250–323)	287 ± 21 (246–328)	285 ± 24 (238–333)	284 ± 29 (227–342)	0.38
IVRT, ms	81 ± 12 (57–104)	89 ± 14 (61–116)	104 ± 17 (70–138)	113 ± 18 (76–149)	< 0.001	86 ± 14 (58–113)	97 ± 15 (66–127)	108 ± 17 (75–142)	116 ± 17 (82–149)	< 0.001
MPI	0.41 ± 0.06 (0.29–0.53)	0.44 ± 0.07 (0.30–0.58)	0.49 ± 0.08 (0.33–0.65)	0.54 ± 0.11 (0.32–0.76)	< 0.001	0.43 ± 0.07 (0.29–0.57)	0.47 ± 0.07 (0.33–0.61)	0.52 ± 0.09 (0.34–0.70)	0.55 ± 0.09 (0.37–0.73)	< 0.001

Data are reported as mean ± SD with corresponding 95% prediction intervals

*IVCT* isovolumic contraction time, *IVRT* isovolumic relaxation time, *LVET* left ventricular ejection time, *MPI* myocardial performance index

### Normal reference ranges of the cardiac time intervals corrected for heart rate

Table 4 shows the developed regression equations relating heart rate to each cardiac time interval and normal reference ranges of the cardiac time intervals including 95% PI in the entire study population and stratified by sex. In females, the regression equation for IVCT was found to be 0.18 × HR + IVCT and the IVCT corrected for heart rate was found to be 52 ± 9 ms (95% PI 34–70 ms). In males, the regression equation for IVCT was found to be 0.15 × HR + IVCT and the IVCT corrected for heart rate was found to be 48 ± 10 ms (95% PI 28–68 ms). In females, the regression equation for LVET was found to be 1.51 × HR + LVET and the LVET corrected for heart rate was found to be 393 ± 16 ms (95% PI 361–423 ms). In males, the regression equation for LVET was found to be 1.38 × HR + LVET and the LVET corrected for heart rate was found to be 372 ± 17 ms (95% PI 340–406 ms). In females, the regression equation for IVRT was found to be 0.30 × HR + IVRT and the IVRT corrected for heart rate was found to be 113 ± 19 ms (95% PI 77–150 ms). In males, the regression equation for IVRT was found to be 0.19 × HR + IVRT and the IVRT corrected for heart rate was found to be 111 ± 19 ms (95% PI 74–149 ms).

**Table 4:** Normal values of the cardiac time intervals corrected for heart rate and regression equations stratified by sex

Cardiac time intervals	All *n* = 1954		Females *n* = 1201		Males *n* = 753		*p* value for sex difference
	Regression equation	Normal values	Regression equation	Normal values	Regression equation	Normal values	
IVCT, ms	0.15 × HR + IVCT	49 ± 10 (30–68)	0.18 × HR + IVCT	52 ± 9 (34–70)	0.15 × HR + IVCT	48 ± 10 (28–68)	< 0.001
LVET, ms	1.4 × HR + LVET	381 ± 17 (347–415)	1.51 × HR + LVET	393 ± 16 (361–423)	1.38 × HR + LVET	372 ± 17 (340–406)	< 0.001
IVRT, ms	0.27 × HR + IVRT	113 ± 19 (76–151)	0.30 × HR + IVRT	113 ± 19 (77–150)	0.19 × HR + IVRT	111 ± 19 (74–149)	< 0.001

Data are reported as mean ± SD with corresponding 95% prediction intervals

*IVCT* isovolumic contraction time, *IVRT* isovolumic relaxation time, *LVET* left ventricular ejection time, *HR* heart rate

**Fig. 3:**
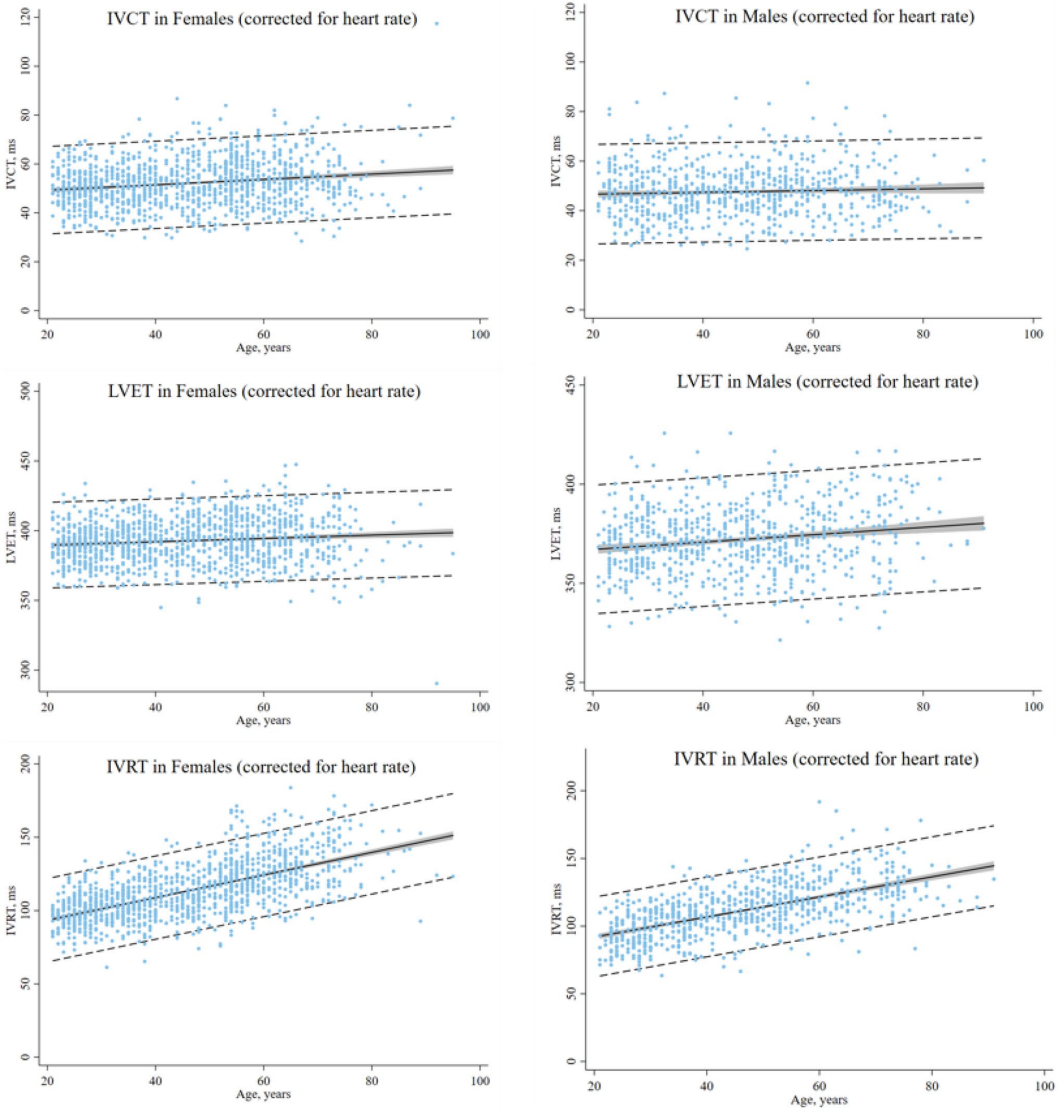
Normal reference ranges of the IVCT, LVET, IVRT corrected for heart rate according to sex and age. The regression line is in black. The 95% confidence interval is in gray. The 95% prediction interval is the dotted black line. *IVCT* isovolumic contraction time, *IVRT* isovolumic relaxation time, *LVET* left ventricular ejection time

Tables 5 and 6 show regression equations relating heart rate to each cardiac time interval and normal reference ranges including 95% PI stratified by sex and age. Figure 3 illustrates the association between the cardiac time intervals corrected for heart rate and age in females and males.

**Table 5:** Normal values of the cardiac time intervals corrected for heart rate and regression equation across age in females

Age range, years	Females								
	Regression equation	20–34 *n* = 348	Regression equation	35–49 *n* = 331	Regression equation	50–64 *n* = 364	Regression equation	> 65 *n* = 158	*p* value for trend
IVCT, ms	0.23 × HR + IVCT	53 ± 8 (38–68)	0.22 × HR + IVCT	54 ± 9 (37–72)	0.22 × HR + IVCT	56 ± 9 (38–75)	0.11 × HR + IVCT	49 ± 11 (27–72)	< 0.001
LVET, ms	1.43 × HR + LVET	384 ± 13 (358–410)	1.37 × HR + LVET	384 ± 14 (355–412)	1.56 × HR + LVET	399 ± 16 (367–430)	1.83 × HR + LVET	415 ± 20 (376–455)	0.08
IVRT, ms	0.37 × HR + IVRT	105 ± 11 (82–127)	0.41 × HR + IVRT	115 ± 13 (88–141)	0.26 × HR + IVRT	120 ± 17 (87–153)	0.53 × HR + IVRT	149 ± 19 (114–184)	< 0.001

*IVCT* isovolumic contraction time, *IVRT* isovolumic relaxation time, *LVET* left ventricular ejection time, *HR* heart rate

**Table 6:** Normal values of the cardiac time intervals corrected for heart rate and regression equation across age in males

Age range, years	Males								
	Regression equation	20–34 *n* = 207	Regression equation	35–49 *n* = 235	Regression equation	50–64 *n* = 199	Regression equation	> 65 *n* = 112	*p* value for trend
IVCT, ms	0.05 × HR + IVCT	41 ± 11 (20–63)	0.30 × HR + IVCT	56 ± 9 (38–74)	0.19 × HR + IVCT	51 ± 11 (31–72)	0.01 × HR + IVCT	39 ± 10 (20–58)	0.55
LVET, ms	1.17 × HR + LVET	356 ± 15 (326–386)	1.25 × HR + LVET	363 ± 15 (334–393)	1.67 × HR + LVET	391 ± 17 (358–425)	1.73 × HR + LVET	401 ± 19 (363–439)	0.38
IVRT, ms	0.28 × HR + IVRT	102 ± 14 (76–129)	0.56 × HR + IVRT	131 ± 14 (103–159)	0.24 × HR + IVRT	124 ± 17 (90–157)	0.84 × HR + IVRT	172 ± 17 (139–205)	< 0.001

Data are reported as mean ± SD with corresponding 95% prediction intervals

*IVCT* isovolumic contraction time, *IVRT* isovolumic relaxation time, *LVET* left ventricular ejection time, *HR* heart rate

### Normal reference ranges of the cardiac time intervals stratified by LAVI, GLS and PALS

In a sensitivity analysis, we included a total of 1954 healthy participants where measurements of the cardiac time intervals and LAVI were available. The IVCT was significantly increased in participants with abnormal LAVI than those with normal LAVI. The LVET, IVRT and MPI did not differ significantly with regard to the size of LAVI (Supplemental Table S1).

In another sensitivity analysis, we included a total of 1903 healthy participants where measurements of the cardiac time intervals and GLS were available. All the cardiac time intervals differed significantly between participants with normal GLS and abnormal GLS. In participants with abnormal GLS, the IVCT was increased, the LVET was decreased, and the IVRT and MPI were increased (Supplemental Table S2). Additionally, in another sensitivity analysis we included a total of 1785 healthy participants with available measurements of the cardiac time intervals and PALS. All the cardiac time intervals differed significantly between participants with normal PALS and abnormal PALS. The IVCT was increased, the LVET was decreased, and the IVRT and MPI were increased in participants with abnormal PALS (Supplemental Table S3).

### Normal reference ranges of the cardiac time intervals according to hypertension and atrial fibrillation

In a secondary analysis to assess the normal values in participants with hypertension or atrial fibrillation (AF), we included participants regardless of health status (*n* = 4374). The IVCT did not differ between participants with hypertension and without hypertension. In participants with hypertension, the LVET was decreased, while the IVRT and MPI were increased (Supplemental Table S4). In participants with AF, the IVCT was increased, the LVET was decreased and the MPI increased. The IVRT did not differ between participants with and without AF (Supplemental Table S5).

## Discussion

This is the largest study providing normal reference ranges for the IVCT, LVET, IVRT and MPI in participants free of cardiovascular diseases and risk factors. In the current study, we report several relevant findings. (1) We established normal reference ranges including 95% PI for IVCT, LVET, IVRT and MPI in the entire study population and across four age categories for each sex. (2) We demonstrated that duration of all the cardiac time intervals differed significantly between females and males. (3) Additionally, we demonstrated how the duration of the cardiac time intervals varies with increasing age for each sex. (4) We developed regression equations relating the heart rate to IVCT, LVET and IVRT in the entire population and across four age categories for each sex.

Few studies have reported normal reference ranges for the cardiac time intervals. Weissler et al. established normal reference ranges for the systolic time intervals including IVCT and LVET [6]. The study was based on a total of 211 healthy participants (121 males and 90 females) aged 19–65 years. However, the definition of the healthy participants was not specified in the study. The IVCT and LVET were measured using simultaneous recordings of the electrocardiogram, the phonocardiogram and the carotid arterial pulse. These measurements were performed with the participants in supine position and fasting [6]. The normal reference ranges for IVCT and LVET were corrected for heart rate and reported as mean ± SD. The normal reference ranges of LVET were reported to be 418 ± 10 ms in females and 413 ± 10 ms in males, while the normal reference ranges of IVCT were reported to be 39 ± 9 ms in females and 33 ± 10 ms in males. In the current study, we found that the normal reference ranges of LVET were 393 ± 16 ms (95% PI 361–423 ms) in females and 372 ± 17 ms (95% PI 340–406 ms), while the normal reference ranges of IVCT were found to be 52 ± 9 ms (95% PI 34–70 ms) in females and 48 ± 10 ms (95% PI 28–68 ms). The difference in the normal reference ranges between the current study and the study by Weissler et al. in 1968 may be due to the different methods of measuring the cardiac time intervals. Furthermore, the prevalence of cardiovascular diseases in the general population has changed, which also may contribute to the difference in the normal reference ranges.

Another study by Biering-Sørensen et al. developed normal reference ranges for all the cardiac time intervals including MPI [8]. This study included participants from the fourth CCHS spanning from 2001 to 2003. A total of 974 participants (553 females and 421 males) without hypertension, diabetes, AF, heart failure and ischemic heart disease were included. The cardiac time intervals were measured by TDI M-mode. The normal reference ranges for the cardiac time intervals were reported as mean ± SD. However, the study did not report normal reference ranges for the cardiac time intervals corrected for heart rate. The authors found that all the cardiac time intervals except IVRT differed significantly between females and males. However, in the current study we found that all the cardiac time intervals including IVRT differed significantly between females and males. This is in accordance with the existing knowledge about sex-specific differences in cardiac structure and function [15, 16]. With increasing age, the IVCT increased in females, but not in males. It has previously been shown that both advanced age and female sex are associated with an increase ventricular systolic and diastolic stiffness even in the absence of cardiovascular diseases [17]. Therefore, we speculate that the increased ventricular systolic stiffness could potentially lead to the increase in IVCT observed in females.

We found that the IVRT increased with increasing age in both sexes, which is in agreement with previous study findings [18, 19]. Bukachi et al. reported that the IVRT increased with increasing age in participants from the general population (*n* = 128) aged 25–88 years [19].

In healthy subjects (*n* = 43), Reant et al. have reported the normal reference ranges for the LVET as mean ± SD and developed a regression equation relating the heart rate to the LVET [20]. However, this study was not dedicated to establish normal reference ranges. The healthy subjects were comprised of a group without heart disease, diabetes, or hypertension and with normal echocardiography and electrocardiogram. The LVET was measured using pulsed-wave Doppler [20]. The authors developed the following regression equation relating the heart rate to the LVET: (LVET = 1.5 × HR + LVET). Similarly, in our study we developed the following equation for the entire study population (LVET = 1.4 × HR + LVET).

In the current study, we found that the LVET did not increase in neither females nor males. In accordance with our findings, it has previously been demonstrated that in 278 healthy participants aged 23–80 years, the LVET did not increase [21]. In the fourth CCHS, it was reported that the LVET decreased with increasing age; however, this decline was not statistically significant [8]. In contrast to these two studies, it has been shown that in 512 elderly participants aged 60–90 years, the LVET increased with aging [7]. The elderly participants with heart failure and those who used digitalis were excluded. However, participants with other cardiovascular diseases and risk factors were not excluded, which can explain the differences between this study and our study findings regarding LVET. Hence, the increase in LVET may be due to other cardiovascular diseases and not only increasing age. Furthermore, the study focused primarily on elderly participants, whereas our study assessed the effect of age on the cardiac time intervals across four age categories.

When comparing our study with all the above-mentioned studies, we included a lager study sample of participants (*n* = 1969) free of cardiovascular diseases and risk factors. Additionally, our study sample was comprised of participants with a wide spectrum of ages spanning from 21 to 97 years, which make it possible to develop normal reference ranges for the cardiac time intervals across four age categories for each sex. The above-mentioned studies only report the normal reference ranges as mean ± SD. However, in our study the developed normal reference ranges are reported as mean ± SD with corresponding 95% PI.

The finding that only IVCT was increased in participants with abnormal LAVI may due to the fact that during the IVCT, the left atrial pressure (LA) increases. Hence, an increased duration of the IVCT may lead to higher LA pressure. Thus, increased LA pressure may result in an enlargement of the left atrium. In a previous study from the fourth round of the CCHS including 1915 participants from the general population, we found that the IVCT was an independent predictor of AF and complicated AF defined as occurrence of either stroke or heart failure following the diagnosis of AF. No associations between LVET, IVRT, MPI, and AF remained significant after multivariable adjustment [22].

The normal values of the cardiac time intervals differed significantly between participants with normal GLS and abnormal GLS, and normal PALS and abnormal PALS, respectively. We excluded participants with cardiovascular diseases and risk factors to ensure a healthy study sample. However, the difference in normal values of the cardiac time intervals in participants with abnormal GLS or PALS may be due to the fact that both GLS and PALS are impaired before the cardiovascular diseases occur. We have previously shown that both GLS and PALS are independent predictors of cardiovascular morbidity and mortality in the general population [23–25].

In participants with hypertension, the LVET was significantly decreased, while the IVRT and MPI were significantly increased. Our findings are in agreement with previous studies that have demonstrated that the LVET was decreased, while the IVRT was increased in hypertensive patients [26–28].

In participants with AF, we found that the IVCT was increased, the LVET was decreased and the MPI increased. The IVRT did not differ between participants with and without AF. Our findings are in accordance with a previous study that have demonstrated that the LVET was decreased in patients with AF, while the pre-ejection time (PEP) including the IVCT increased [29].

Using the TDI M-mode method, the cardiac time intervals can be obtained with greater precision and better reproducibility as it is much easier to identify the clear color shifts caused by mitral valve movements throughout the cardiac cycle. By contrast, it can be difficult to accurately define the cardiac time intervals from velocity curves, when using the conventional pulsed-wave Doppler method [4, 5, 23, 30]. Moreover, the improvement of the TDI M-mode method may be due to the very high frame rate, typically from 160 to 200 frames per second, while the conventional pulsed-wave Doppler method uses a lower frame rate [31]. Additionally, when using the TDI M-mode method, the cardiac time intervals can be obtained regardless of heart rhythm, whereas the conventional method cannot assess the cardiac time intervals in patients with AF [12]. The cardiac time intervals can be obtained using the conventional pulsed-wave Doppler method as previously described by Tei and colleagues [32]. The interval between the onset and end of mitral inflow is equal to the sum of the IVCT, LVET, and IVRT (interval *b*). The duration of the LV outflow velocity profile is equal to the LVET (interval *a*). By subtracting b from a, the sum of the IVCT and IVRT can be obtained [32]. In patients with AF, there is no definite A wave or *a*′ wave due to lack of organized atrial activity [33]. Hence, the onset and end of mitral inflow and thereby the IVCT and IVRT cannot be assessed accurately due to the absence of A wave.

There is an increasing interest in the cardiac time intervals, since a novel drug (Omecamtiv mecarbil (OM)) acts specifically by increasing the LVET in patients with heart failure with reduced ejection fraction (HFrEF) [34]. Treatment with OM has very recently shown to improve outcomes in patients with HFrEF [35]. Therefore, it is of great clinical importance to develop age- and sex-based normal reference ranges for the cardiac time intervals. However, future studies are required to determine whether there is a desirable target of the cardiac time intervals in patients with HFrEF.

### Study limitations

There are potential limitations to the current study. The CCHS is primarily composed of Caucasians, which limits the generalizability of our developed normal reference ranges for the cardiac time intervals to other populations with another composition. The cardiac time intervals can be measured by different methods including pulsed-wave Doppler, pulsed-wave TDI and TDI M-mode method [2]. These different methods of obtaining the cardiac time intervals may lead to different normal values. Therefore, our established normal reference ranges may not be generalizable to the cardiac time intervals obtained by other methods. Hence, future studies are required to assess the normal values of the cardiac time intervals for each method. In the current study, we don’t have data available regarding measurements of the cardiac time intervals using the pulsed-wave Doppler method. Therefore, we could not compare our findings based on the TDI M-mode method with measurements of the cardiac time intervals based on the conventional method from this cohort. Due to the high prevalence of cardiovascular diseases and risk factors among males, we excluded a higher proportion of males than females. Hence, our study sample was not fully representative of the general population.

A high prevalence of cardiovascular diseases and risk factors was among the participants aged > 65 years. Since we excluded participants with cardiovascular diseases and risk factors to ensure a healthy study sample, our group of participants aged > 65 years was small. Therefore, the assessment of the association of the cardiac intervals with age is limited. However, this group was larger, when comparing it to other studies that developed normal reference ranges for the cardiac time intervals.

## Conclusion

In the current study, we established normal age- and sex-based reference ranges for the cardiac time intervals. Further, we developed both regression equations and age- and sex-based reference ranges relating the heart rate to the cardiac time intervals. We found that the duration of the cardiac time intervals differed significantly between females and males. With increasing age, the IVCT increased in females, but not in males. The LVET did not change in both sexes, while the IVRT increased in both sexes with increasing age. This emphasizes the need for age- and sex-based normal reference ranges for the cardiac time intervals.

## Supplementary Information

Below is the link to the electronic supplementary material.

Supplementary file1 (DOCX 23 kb)
