# Supplementary material for: Age- and sex-based normal reference ranges of the cardiac time intervals: the Copenhagen City Heart Study
Source: Clin Res Cardiol. 2023 Jul 31;114(4):430–42. doi: 10.1007/s00392-023-02269-2 (PMC11946970; doi:10.1007/s00392-023-02269-2)
Supplement: Supplementary file 1 — Supplementary file1 (DOCX 23 kb) [file 392_2023_2269_MOESM1_ESM.docx]

**SUPPLEMENTAL MATERIAL**

Age-and Sex-Based Normal Reference Ranges of the Cardiac Time Intervals: The Copenhagen City Heart Study

Alia Saed Alhakak, MD, PhD^1^; Flemming Javier Olsen, MD, PhD^1^; Kristoffer Grundtvig Skaarup, MD^1^; Mats Christian Højbjerg Lassen, MD^1^; Niklas Dyrby Johansen, MD^1^; Peter Godsk Jørgensen, MD, PhD^1,2^; Ulrik Abildgaard, MD, DMSc^1^; Gorm Boje Jensen, MD, DMSc^2^; Peter Schnohr, MD, DMSc^2^; Peter Søgaard, MD, DMSc^2,3,4^; Rasmus Møgelvang, MD, PhD^2,5,6,7^; Tor Biering-Sørensen, MD, MSc, MPH, PhD^1,2,8^

1) Department of Cardiology, Herlev and Gentofte Hospital, University of Copenhagen, Copenhagen, Denmark

2) The Copenhagen City Heart Study, Bispebjerg and Frederiksberg Hospital, University of Copenhagen, Copenhagen, Denmark

3) Department of Cardiology, Aalborg University Hospital, Aalborg, Denmark

4) Department of Clinical Medicine, University of Aalborg, Aalborg, Denmark

5) Department of Cardiology, The Heart Center, Rigshospitalet, University of Copenhagen, Copenhagen, Denmark

6) Institute of Clinical Medicine, Faculty of Health and Medical Sciences, University of Copenhagen, Copenhagen, Denmark

7) Cardiovascular Research Unit, University of Southern Denmark, Odense, Denmark

8) Department of Biomedical Sciences, Faculty of Health and Medical Sciences, University of Copenhagen, Copenhagen, Denmark

**Corresponding author:**

Alia Saed Alhakak, MD, PhD

Cardiovascular Non-Invasive Imaging Research Laboratory

Department of Cardiology, Herlev and Gentofte Hospital, University of Copenhagen, Denmark

Gentofte Hospitalsvej 1, Post 835, 2900, Hellerup, Copenhagen, Denmark

Telephone: +45 60686063, fax: +45 39777381

Email: [aliasaed@hotmail.com](mailto:aliasaed@hotmail.com)

**Running title:** Normal values of the cardiac time intervals

**Table S1:** Normal values of the cardiac time intervals stratified by LAVI

| **Cardiac time intervals** | **Normal LAVI**  **(≤34mL/m^2^)**  **n=1818** | **Abnormal LAVI**  **(>34mL/m^2^)**  **n=136** | **P-value for difference** |
| --- | --- | --- | --- |
| **IVCT, ms** | 40±10(20-59) | 42±11 (19-65) | 0.005 |
| **LVET, ms** | 292±22 (248-335) | 295±25 (247-344) | 0.067 |
| **IVRT, ms** | 96±19 (59-134) | 99±20 (59-138) | 0.167 |
| **MPI** | 0.47±0.09 (0.29-0.65) | 0.48±0.10 (0.28-0.68) | 0.141 |

IVCT, isovolumic contraction time; IVRT, isovolumic relaxation time; LAVI, left atrial volume index; LVET, left ventricular ejection time; MPI, myocardial performance index

**Table S2:** Normal values of the cardiac time intervals stratified by GLS

| **Cardiac time intervals** | **Normal GLS**  **(≥15.8%)**  **n=1859** | **Abnormal GLS**  **(<15.8%)**  **n=44** | **P-value for difference** |
| --- | --- | --- | --- |
| **IVCT, ms** | 40±10(20-59) | 46±11 (24-69) | <0.001 |
| **LVET, ms** | 293±22 (250-336) | 269±23 (224-314) | <0.001 |
| **IVRT, ms** | 96±19 (59-133) | 112±22 (68-156) | <0.001 |
| **MPI** | 0.46±0.09 (0.28-0.64) | 0.59±0.11 (0.37-0.81) | <0.001 |

GLS, Global longitudinal strain; IVCT, isovolumic contraction time; IVRT, isovolumic relaxation time; LVET, left ventricular ejection time; MPI, myocardial performance index

**Table S3:** Normal values of the cardiac time intervals stratified by PALS

| **Cardiac time intervals** | **Normal PALS**  **(≥23%)**  **n=1729** | **Abnormal**  **(<23%)**  **PALS**  **n=56** | **P-value for difference** |
| --- | --- | --- | --- |
| **IVCT, ms** | 39±10(21-58) | 42±14 (14-71) | 0.036 |
| **LVET, ms** | 293±22 (249-336) | 283±28 (227-339) | 0.0012 |
| **IVRT, ms** | 96±19 (59-133) | 110±14 (82-138) | <0.001 |
| **MPI** | 0.46±0.09 (0.28-0.64) | 0.54±0.11 (0.32-0.76) | <0.001 |

IVCT, isovolumic contraction time; IVRT, isovolumic relaxation time; LVET, left ventricular ejection time; MPI, myocardial performance index; PALS, peak atrial longitudinal strain

**Table S4:** Normal values of the cardiac time intervals stratified by hypertension

| **Cardiac time intervals** | **Hypertension**  **n=2278** | **No hypertension**  **n=2096** | **P-value for difference** |
| --- | --- | --- | --- |
| **IVCT, ms** | 41±12 (16-65) | 40±10 (20-60) | 0.069 |
| **LVET, ms** | 287±31 (227-347) | 291±23 (246-337) | <0.001 |
| **IVRT, ms** | 110±22 (66-154) | 97±20 (58-137) | <0.001 |
| **MPI** | 0.53±0.12 (0.29-0.77) | 0.47±0.10 (0.27-0.67) | <0.001 |

IVCT, isovolumic contraction time; IVRT, isovolumic relaxation time; LVET, left ventricular ejection time; MPI, myocardial performance index

**Table S5:** Normal values of the cardiac time intervals stratified by atrial fibrillation

| **Cardiac time intervals** | **Atrial fibrillation**  **n=189** | **No atrial fibrillation**  **n=4185** | **P-value for difference** |
| --- | --- | --- | --- |
| **IVCT, ms** | 45±16 (14-76) | 40±11 (18-62) | <0.001 |
| **LVET, ms** | 272±42 (188-355) | 290±26 (238-342) | <0.001 |
| **IVRT, ms** | 107±29 (50-164) | 104±29 (61-147) | 0.068 |
| **MPI** | 0.58±0.18 (0.23-0.93) | 0.50±0.11 (0.25-0.69) | <0.001 |

IVCT, isovolumic contraction time; IVRT, isovolumic relaxation time; LVET, left ventricular ejection time; MPI, myocardial performance index
